# Whole-Body Cryostimulation, a Complementary Treatment for Phantom Limb Syndrome: Preliminary Evidence from a Case Study

**DOI:** 10.3390/medicina60010022

**Published:** 2023-12-22

**Authors:** Paolo Piterà, Isabella Springhetti, Angelo Alito, Federica Verme, Jacopo Maria Fontana, Paolo Capodaglio

**Affiliations:** 1 Research Laboratory in Biomechanics, Rehabilitation and Ergonomics, IRCCS, Istituto Auxologico Italiano, San Giuseppe Hospital, 28824 Verbania, Italy; i.springhetti@auxologico.it (I.S.); f.verme@auxologico.it (F.V.); j.fontana@auxologico.it (J.M.F.); p.capodaglio@auxologico.it (P.C.); 2 Department of Biomedical, Dental Sciences and Morphological and Functional Images, University of Messina, 98125 Messina, Italy; alitoa@unime.it; 3 Department of Surgical Sciences, Physical Medicine and Rehabilitation, University of Torino, 10126 Torino, Italy

**Keywords:** whole-body cryostimulation, phantom limb pain, phantom limb syndrome, neuropathic pain, central sensitization

## Abstract

Phantom limb pain (PLP) is a challenging condition affecting a significant proportion of amputees. In this article, we describe the case of a 54-year-old Paralympic athlete with phantom limb syndrome following right leg amputation and widespread sports-related enthesitic pain who underwent a whole-body cryostimulation (WBC) cycle, an emerging treatment known for its rapid pain-relieving and anti-inflammatory effects. Assessments were conducted before and after a 10-session WBC cycle, including pain and quality of life assessment and use of medications. A substantial reduction in enthesitic pain, PLP intensity, paresthesia, and tingling related to atmospheric events and improved function and quality of life were reported after the WBC cycle and lasted for two weeks. One month after WBC, the enthesitic pain following sports activity and PLP gradually returned, but with lesser intensity. Similarly, the stump’s sensitivity to atmospheric changes returned, but with lower frequency. Pain at night remained lower than before WBC, with significantly improved quality of sleep. This case study suggests that WBC could be a valuable adjuvant treatment for alleviating PLP. Controlled studies are warranted to validate the findings of this case report and elucidate the mechanisms underlying the positive effects of WBC in this condition.

## 1. Introduction

Phantom limb pain (PLP) is clinically defined as the perception of pain or discomfort in a limb that no longer exists [1] and is commonly observed as a consequence of amputation, affecting as many as 80% of individuals who undergo this surgical procedure [2]. The etiology of PLP appears to be predominantly linked to central neural changes; however, peripheral and psychological factors may also contribute to its manifestation. 

The typical pain experienced in PLP is attributed to a dysfunction in the transmission of pain signals within the nervous system [3]. Indeed, although the mechanisms underlying PLP remain unclear, it is known that sensitized and reorganized nerve endings and cell bodies within the peripheral limb affect the CNS, causing changes in somatosensory processing pathways [3]. This process can result in the formation of neuromas, neoformations of nerve fibers of a pathological nature, characterized by abnormal activity, either spontaneously or as a result of mechanical stimulation of the stump. The pain may be severe at first, tending to ease over time; it is usually intermittent, but in some cases, it can last for days or, in some people, become chronic and persist for years [4]. Phantom sensation is a general term for a wide variety of symptoms [5]. These sensations encompass positional, morphological, or kinetic attributes of the phantom, as well as pain, warmth, cold, itching, tingling, electric impulses, and other paraesthesias [6]. After limb amputation, patients often report that the deafferented part of the body remains perceptually intact, even in terms of pain or other sensations. This pain tends to be more intense in the distal parts, and the characteristics of these sensations are often described as wounding, stabbing, throbbing, burning, aching, pinching, stinging, and compressing [2,5].

Phantom pain can be associated with specific limb positions or movements and can be provoked or aggravated by various physical factors, such as changes in atmospheric conditions or pressure on the limb, and psychological factors, such as emotional stress [2,7]. 

This type of chronic pain is often combined with symptoms such as sleep disturbances, anxiety, and depression [8]. It is known that those emotional triggers contribute to the persistence and exacerbation of PLP, leading to central sensitization, a pathological condition characterized by increased neuronal activity, lowering of the neuron activation threshold, and spreading to other sites [9]. The increased activity of peripheral nociceptors leads to changes in the structure of dorsal horn neurons, with increased responsiveness to stimuli and reduced inhibitory activity. In addition, peripheral nerve injury can result in degeneration of C-fibers in the lamina II of the spinal cord and subsequent colonization of A-beta fibers, which are responsible for the transmission of non-painful stimuli, leading to misinterpretation. At the brain level, reorganization of the somatosensory cortex occurs as a result of the discrepancy between motor commands and proprioceptive feedback from the missing limb [10]. Several studies have found a strong correlation between the degree of brain reorganization described above and the intensity of PLP [2,7].

The treatment of PLP is complex, and recent studies have found limited evidence regarding the choice of pharmacological treatment. Some of these studies, however, have observed the effectiveness of morphine and gabapentin in reducing pain intensity while emphasizing the presence of side effects that limit their usability. Given the complexity of the pathological mechanism underlying chronic pain and the multitude of associated symptoms, additional therapeutic options beyond pharmacological treatment are deemed necessary, as pharmacotherapy alone is often insufficient [11].

Whole-body cryostimulation (WBC) is a therapeutic procedure involving exposure of the entire body to extremely cold air at temperatures as low as −140 °C for 2–3 min. Widely recognized in the realm of sports for its well-documented pain-relieving and anti-inflammatory properties [12], WBC is increasingly garnering attention within the clinical domains. In fact, several studies have shown beneficial effects in patients with various conditions characterized by chronic pain, fatigue, and inflammation, such as obesity, fibromyalgia, and neurological disorders [13,14,15,16]. WBC can slow nerve conduction velocity, modulate the autonomic nervous system, stimulate endorphin secretion from the brain, and induce exercise-mimicking molecular anti-inflammatory effects [17], with direct implications for pain control [18]. Therefore, we hypothesized that WBC may be beneficial in relieving symptoms of patients with PLP. To the best of our knowledge, no studies to date have addressed this specific issue.

## 2. Methods

### 2.1. Brief Case Description

Here we report the case of R.N., a 54-year-old male Paralympic athlete with phantom limb syndrome secondary to right lower limb amputation in 2004. The patient was referred as an outpatient to the Rehabilitation Unit of Istituto Auxologico Italiano, Piancavallo (VB), Italy, in August 2023 for a cycle of ten WBC sessions prescribed by a physiatrist (PC).

### 2.2. Clinical History

At the age of 15 years, he underwent open heart surgery for a congenital atrial septal defect. At the age of 20 years, following a car accident, he suffered an incomplete D7 spinal cord injury, which was managed conservatively with an orthosis, and then he was bedridden for 3 months. He resumed daily activities with a spinal brace. At the age of 33, while working with a chainsaw, he suffered a muscle injury to the rectus muscle in the center of his thigh, which required 80 stitches, with no other consequences. At the age of 35, while working on the family farm, the patient suffered an injury in which his right lower limb was caught in the drive shaft of a tractor, which tore off the limb in the upper third of the tibia, causing it to be amputated. The patient was taken to the emergency department and underwent amputation at the mid-thigh of the right leg. The contralateral left lower limb also suffered muscle loss in the thigh and lower third of the leg, but subsequently recovered without residual anatomical or functional deficits. In the immediate post-operative period, pain was managed with opioid infusion, ketorolac, and centrally acting analgesics (gabapentin). PLP started soon after the amputation. Prosthetic fitting began 3 months after the incident. Upright posture and movement were restored in about 1 month. Three months after the accident, the patient was able to walk with a temporary mechanical prosthesis while waiting for an electronic prosthesis, which was provided after 7 months. One year later, he resumed his previous job on the farm, but performance was below pre-injury levels. PLP lasted 18 months and gradually decreased in intensity. A remitting painful sensation persisted, typically lasting 4–5 days and triggered by weather changes. At the age of 45, he was abusing psychotropics (amphetamine), pharmacological substances (citalopram), and alcohol and was diagnosed with myocarditis. Two years later, he managed to wean off the addictions and started a canoeing and kayaking sports program for people with disabilities. He also changed his job, becoming a safety officer for a company. Over time, he achieved success in competitive sports. He is currently a multiple Italian paracanoe champion and two-time world champion in para-rafting with the national amputee team.

### 2.3. Clinical Examination

On examination, the patient presented with PLP and diffuse sports-related enthesitic pain in the right shoulder and right groin and chronic pain in the lower lumbar region radiating to the left hip, gluteus, and anterior thigh. He reported persistent paresthesia or tingling, similar to numbness, occasionally accompanied by excruciating pain attacks described as “shocks” or “electric jolts”. This caused considerable discomfort and anger, which was difficult to manage since no effective remedies had been found over the years. This affected his emotional state and overall quality of life. The pain episodes typically lasted 4–5 days and were usually triggered by sudden temperature or weather changes. The patient also reported severe pain at night, which severely affected his mood and sleep quality. Subjectively, he described the pain as “coming from the brain”, as if “the pain is in the brain because that’s where the suffering comes from”.

### 2.4. Symptoms Assessment

The clinical evaluations included assessments conducted both before and after the WBC cycle. Pain levels were assessed using the numeric rating scale (NRS) [19]. To discriminate between neuropathic and nociceptive pain, the Leeds Assessment of Neuropathic Symptoms and Signs (LANSS) was used [20]. The quality and intensity of pain were analyzed using the short-form McGill Pain Questionnaire 2 (SF-MPQ-2) [21]. Health-related quality of life was evaluated using the short-form Health Survey 36 (SF-36) [22]. Sleep quality was assessed using the Pittsburgh Sleep Quality Index (PSQI) [23]. General subjective well-being was measured using the 5-item World Health Organization Well-Being Index (WHO-5) [24]. Additionally, the severity of depression was determined using the Beck Depression Inventory (BDI) [25]. 

### 2.5. Intervention 

**Figure 1:**
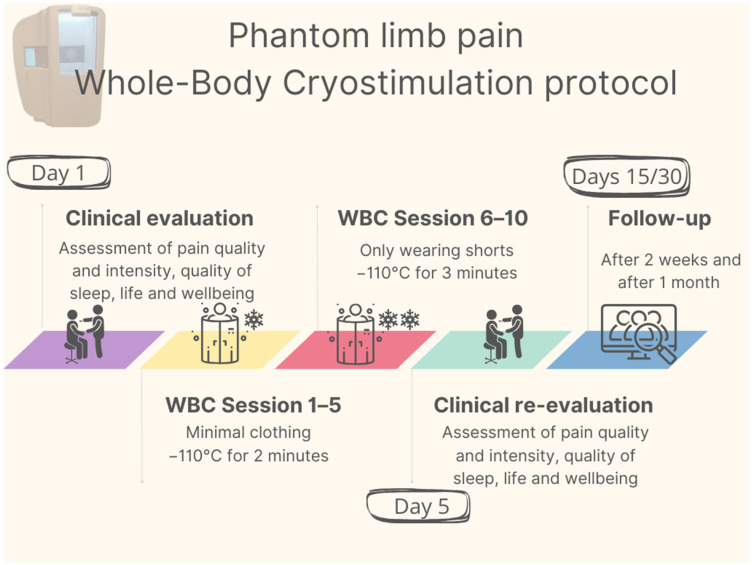
Schematic representation of the WBC rehabilitation protocol.

The intervention consisted of a total of 10 outpatient WBC sessions performed twice a day (at 9 a.m. and 12 p.m.) from 21 to 25 August 2023. No other physiotherapy treatment other than WBC was administered. After confirming the absence of contraindications to WBC treatment according to the Bad Voslau contraindications list [26], the patient underwent an initial 1 min familiarization session, during which he entered the cryochamber (Arctic, CryoScience, Rome, Italy) wearing minimal clothing. He then underwent five 2 min WBC sessions at −110 °C; starting from the sixth session, the treatment duration was increased to 3 min, and the patient was asked to remove his t-shirt to increase the body surface area exposed to the cryogenic cold stimulus. Each treatment was monitored by specially trained personnel. A graphical representation of the protocol is provided in Figure 1.

## 3. Results

At the time of examination, pain sensation, assessed with the LANSS Pain Scale, scored 18/24, suggesting a probable neuropathic mechanism, as a score >12 indicates the presence of PLP.

The results from all the scales and questionnaires administered are shown in Table 1 as pre (before WBC), post (after WBC), and range values.

At the end of the WBC cycle and for the following 2 weeks, the patient reported a noticeable reduction in PLP; in particular, pain exacerbations, sensation of electric current, and tingling during thunderstorms had completely ceased. The present pain intensity (PPI) score improved from “excruciating” before WBC to “discomforting” after WBC. The patient felt elated with the results achieved. The enthesitic pain completely disappeared, with complete recovery of function in all previously affected joints, including during sports activities. He had regained the ability to drive long distances without the pain in the inguinal enthesitis in the healthy limb and reported feeling generally better, sleeping restfully at night, and feeling less anger.

After 2 weeks, the pain gradually reappeared with the same pattern of onset and duration as before the WBC cycle, but with less intensity.

One month after completion of the WBC cycle, the functional and clinical picture had further changed. The symptoms of widespread enthesitis were gradually returning after sports activity, but with less intensity and without the need to stop training. Improvements in the ability to drive pain-free for long distances remained constant. Stump sensitivity to atmospheric changes returned to previous levels but occurred much less frequently. The patient also reported a reduction in analgesic medications and nighttime pain, with a significant improvement in sleep quality compared with before WBC. 

## 4. Discussion

This first case study on the effects of WBC on PLP describes the positive effects on pain, function, and quality of life in a Paralympic amputee athlete who had previously undergone various pharmacological, physical therapy, and physiotherapy treatments without success. Previous “off-label” prescriptions of WBC in specific conditions, such as post-COVID condition [27], have shown positive effects, likely due to the wide range of effects elicited by WBC on pain, fatigue, and mood [15]. Given the multiple mechanisms at the peripheral and central levels implicated in the development of central sensitization characterizing PLP and considering the broad range of possible symptoms, treatment of PLP calls for interventions acting at multiple levels. WBC is a powerful physical modality that generates a thermal shock, which represents an intense physical experience for the patient. The cold stress induces a series of physiological responses affecting the circulatory, nervous, endocrine, and immune systems. WBC could thus be described as an “adaptive therapy” since repeated cryogenic stimuli induce a stress that, by training the homeostatic systems, elicits adaptations that promote the restoration of homeostasis [28]. Activation of the sympathetic nervous system through cold exposure leads to an increase in the release of noradrenaline, a hormone involved in modulating painful stimuli and activating and enhancing descending inhibitory pathways. This influences the pain response by slowing the speed of nerve conduction, in particular the C-fibers, responsible for the transmission of pain signals. In addition, this modulation involves altering neurotransmitters associated with pain signaling, inhibiting sensory receptors and their connections with proprioceptors, and promoting the release of analgesic factors [18]. Due to its action on the biological mechanisms involved in pain perception and modulation, anti-inflammatory properties [29], and positive impact on fatigue, muscular pain, sleep quality, and anxiety and depressive disorders [30], WBC has already been proposed as an adjuvant therapy in conditions where these functions are altered [15]. Our hypothesis was that WBC could provide beneficial effects in a condition like PLP, characterized by an altered perception of pain resulting from pathological sensitization of the central nervous system. The patient reported rapid benefits, not only on PLP and enthesitic pain but also on sleep, quality of life, and well-being, starting from the very first WBC session and persisting for the following 2 weeks. WBC seems to have rapid but short-lasting analgesic effects on PLP, which may in turn suggest that repeated, intermittent WBC sessions, when pain relapses, could be considered to help modulate pain and follow-up symptoms. Despite symptoms relapsing after 2 weeks, albeit with lower intensity, the patient reported unprecedented benefits after WBC, particularly regarding PLP and sleep quality, compared to other previously experienced pharmacological and physical treatments. The patient’s positive emotional involvement in the treatment and his satisfaction with a short, well-tolerated treatment without adverse effects may have played a role in the functional improvements observed, and a placebo effect cannot be ruled out. Controlled studies with larger samples will be needed to confirm these findings and clarify the specific mechanisms of action of WBC in PLP.

## 5. Conclusions

Our preliminary findings, together with the low incidence of adverse events reported in the literature, suggest that WBC could be studied on a larger scale as a potential adjuvant treatment to be used alongside existing therapies for pain conditions characterized by central sensitization. As this is the first case study on the effects of WBC in patients with PLP, further research and larger studies are needed to confirm the results obtained.

## Figures and Tables

**Table 1:** Questionnaire and scales administered before and after the WBC cycle.

Outcome and Range	Pre	Post
NRS (0–10)	7.5	2
SF-MPQ-2 * (0–10)	5.86	6.90
PPI (Phantom limb) (0–10)	8	6
PPI (Enthesitic pain) (0–10)	7	0
SF-36		
Physical functioning	60%	70%
Role limitations due to physical health	75%	100%
Role limitations due to emotional problems	33.3%	100%
Energy/fatigue	50%	55%
Emotional well-being	56%	80%
Social functioning	62.5%	75%
Pain	35%	77.5%
General health	40%	70%
Health change	25%	0%
WHO-5	52%	60%
PSQI (0–21)	21	8
BDI (0–63)	5	3

NRS = numeric rating scale; SF-MPQ-2 = short-form McGill Pain Questionnaire 2, * referring to the month before the WBC cycle and 1 month after; SF-36 = Short Form Health Survey 36; PSQI = Pittsburgh Sleep Quality Index; WHO-5 = 5-item World Health Organization Well-Being Index; BDI = Beck Depression Inventory.

## Data Availability

The data presented in this study are available within the article.
